# Intrusive growth of initials does not affect cambial circumference in *Robinia pseudoacacia*

**DOI:** 10.1038/s41598-022-11272-y

**Published:** 2022-05-06

**Authors:** Adam Miodek, Aldona Gizińska, Wiesław Włoch, Paweł Kojs

**Affiliations:** 1 Polish Academy of Sciences Botanical Garden – Centre for Biological Diversity Conservation in Powsin, Prawdziwka 2, 02-973 Warsaw, Poland; 2 Institute of Biology, University of Opole, Oleska 22, 45-052 Opole, Poland

**Keywords:** Developmental biology, Plant sciences

## Abstract

This study aimed to test the hypothesis whether intrusive growth of initial cells is related to the increase in circumference of *Robinia pseudoacacia* vascular cambium—both qualitatively and quantitatively. The mode of intrusive growth of cambial initial cells was also studied. Samples collected from tree trunks were examined using series of semi-thin transverse sections. Anatomical reconstructions of radial and tangential planes of analysed fragments of cambial tissue were made. Observations and measurements have shown that the intrusive growth of *R*. *pseudoacacia* initial cells does not contribute to an increase in tangential dimension of observed tissue fragments where cell rearrangement occurs. Moreover, initially separated tangential walls of cells (between which cambial initial cell elongates intrusively) are transformed into obliquely oriented walls. These results stand in accordance with a statement that only symplastic growth of initials, not intrusive growth, is responsible for the increase in circumference in all woody plants with the continuous cambial cylinder. Moreover, we managed to capture the moment of transition of initial status from one cell to another for the first time. This phenomenon may be explained on the basis of the system of mechanical stresses operating not only in the secondary meristematic tissue but also in a whole plant organism.

## Introduction

**Figure 1:**
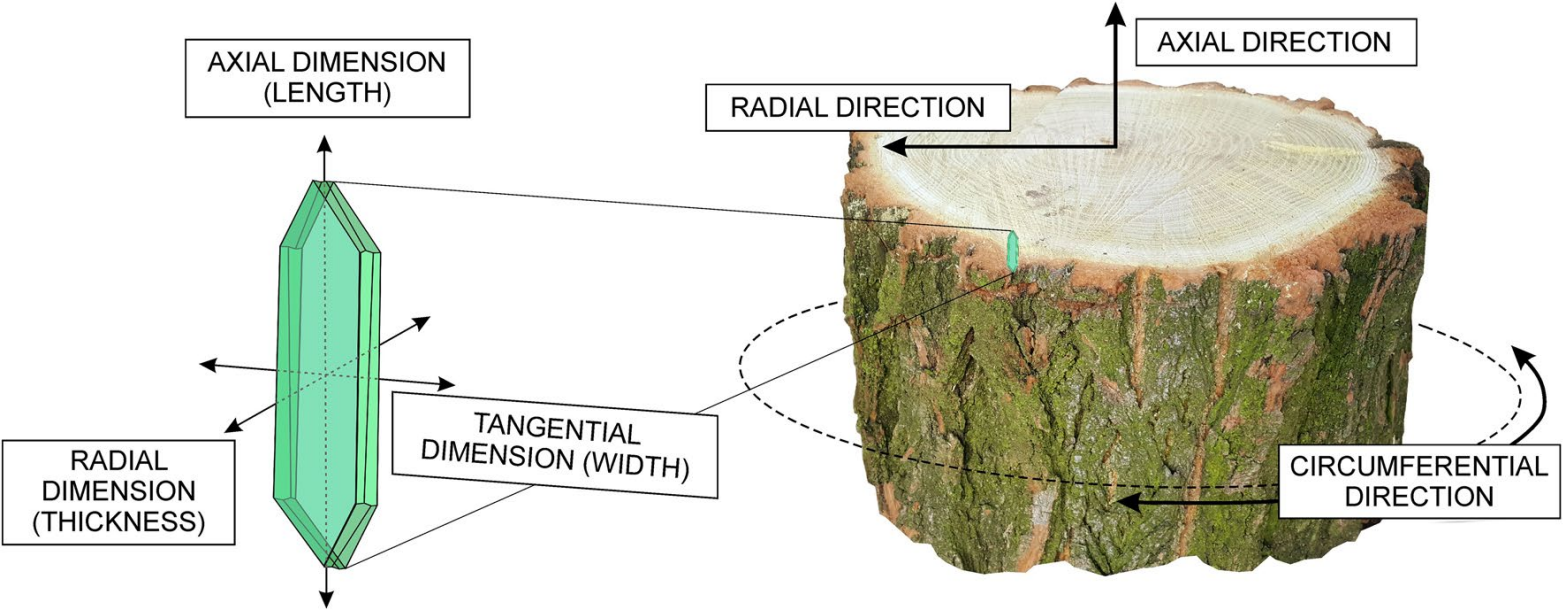
Scheme showing three main directions of cell growth in cylindrical vascular cambium (right), and description of the cell dimensions (left). Descriptions of the cell dimensions illustrate contextual link to the corresponding directions within the tree trunk with cylindrically shaped vascular cambium. Adobe Photoshop CS6 Extended ver.13.0 × 64 (https://www.adobe.com), and CorelDRAW Graphics Suite SE ver. 18.2.0.840 (https://www.coreldraw.com) were used.

In angiosperm trees, a lateral meristem—vascular cambium—is responsible for producing cells of an axial and radial system of secondary xylem and secondary phloem^1,2^. Cells deposited by cambial initials, i.e. cambial derivatives, may undergo tangential, radial, and axial growth (depending on the type of cell) and differentiation followed by the maturation process^3–6^. As a consequence, secondary vascular tissues are formed. Secondary phloem is deposited outwards, and secondary xylem inwards by the cylindrical vascular cambium (Fig. 1).

In angiosperm tree species, two main types of cambial cell arrangement may be distinguished: nonstoried (irregular arrangement of ends of fusiform initials in tangential section), and storied (ends of fusiform initials are arranged in horizontal tiers in tangential section)^7^. Interestingly, these two types of cambium are usually considered in the literature as differing in how they increase their circumference. In some sources, the circumference enlargement of nonstoried cambium is perceived as a result of the intrusive elongation of initial cells which follows oblique anticlinal divisions^1,8–14^. On the other hand, the circumference enlargement of the storied cambium is perceived as a result of coordinated (symplastic) growth which follows longitudinal anticlinal divisions^9–13,15,16^. Thus, the most important difference between these two ways of circumference increase was thought to be in the contribution and role of intrusive growth, i.e. growth which results in the change in contacts between cells^4,13,17–19^. Directional intrusive growth, and other events, such as oblique anticlinal divisions of initials, uniting and splitting of rays^17,19–21^, are components of the rearrangement process of initial cells of the vascular cambium^22–25^.

An argument that two different ways (nonstoried vs. storied cambia) are responsible for an increase in circumference of the vascular cambium, and thus of a whole tree trunk, was based on observations of the range and intensity of intrusive growth and frequency of a given type of anticlinal divisions in these cambia. It was presumed that in nonstoried cambium (in which the range of axial intrusive growth of cells is large), after the occurrence of oblique anticlinal divisions, tips of resultant sister initials grow intrusively between neighbouring initials above and below, and thus increase the area of the vascular cambium^12^. However, some reports have pointed to a different possibility, locating the intrusive growth of initials between tangential cell walls^15,16,24–29^, which implies that intrusive growth does not lead to an increase in circumference of the vascular cambium. Studies challenging the wide-spread view that intrusive growth is associated with growth of cambial circumference have been made on several species including *Lonchocarpus sericeus*^25^, *Picea abies*^30^, *Pinus sylvestris*^15,26–28^, *Tilia cordata*^15,22^, and *Wisteria floribunda*^15,24^. Furthermore, as shown by the thorough mathematical analysis published recently, a tangential symplastic growth of cambial initials alone is entirely sufficient to explain an increase in circumference of meristematic tissue in tree stems^29^. It was highlighted that cambial circumference enlargement is not caused by anticlinal divisions, but symplastic expansion of tangential dimensions of initials, both freshly divided and undivided ones (right after an anticlinal division, resultant sister cells considered together have the same tangential dimension as their mother initial cell)^29^. Due to (1) the newly proposed role of symplastic growth in mathematical modelling of expansion of cambial circumference, and (2) the fact that previously mentioned studies challenging a wide-spread view that intrusive growth leads to an increase in cambial circumference were of qualitative type, a quantitative analysis of influence of intrusive growth on tangential dimension of fragment of tissue within which cambial rearrangement takes place is necessary. The main goal of this study is to further elucidate, both quantitatively and qualitatively, the mechanism of intrusive growth of cambial initial cells on the example of *Robinia pseudoacacia* L.—a tree species of growing economic potential in Europe^31^. It is also one of the most highly invasive alien species threatening biodiversity, which, given the anticipated climate changes, may become an increasing ecological and economic problem^32–35^. During the study it was investigated: (1) whether intrusive growth of initial cells contributes to the tangential enlargement of rearranging fragments of the vascular cambium, and therefore participates in circumferential enlargement of this secondary meristematic tissue, and (2) whether intrusive growth of initial cells in *R. pseudoacacia* vascular cambium occurs between tangential walls of neighbouring cells.

Without a clear understanding of xylogenesis it is impossible to fully comprehend how annual growth rings and wood structure are formed. Knowledge on the xylem formation process that begins in the vascular cambium is crucial, since it is a must-know when it comes to optimisation of wood production. Thus, an attempt to obtain the much needed evidence concerning relation between cambial circumference and intrusive growth of initial cells was made based on the measurements performed in numerous anatomical sections and supported by statistical analysis. For the first time, a quantitative analysis was implemented for this type of investigation, which extends our understanding of the mode and role of intrusive growth of cambial initial cells.

## Materials and methods

### Plant material

*Robinia pseudoacacia* L. is a deciduous tree species with a ring-porous wood structure^1,36^. We chose this species for our study because of the storied structure of its vascular cambial tissue. Due to the highly ordered arrangement of its components, such structure allows for precise anatomical analysis of the course of intrusive growth of initials and wood fibres^37^. Key factor taken into account while choosing this species as the main object of the study was its growing economic importance outside the area of natural distribution^31,38^ due to its high adaptive potential, especially in the context of emerging environmental threats.

### Sample collection and material preparation

Vascular cambium along with the surrounding tissues—secondary xylem and phloem—was collected from two trunks of *R*. *pseudoacacia* (circumference at breast height approx. 9.5 cm and 66.0 cm) growing in the Silesian Botanical Garden, southern Poland (50°10′N, 18°49′E; 331 m above sea level; temperate zone). The samples were collected in the 2013 and 2014 growing seasons (July and September respectively), fixed and stored in mixture of glycerol and ethanol (1:1). The plant material was identified by Dr. Adam Miodek (Polish Academy of Sciences Botanical Garden-Centre for Biological Diversity Conservation in Powsin; University of Opole), and Prof. Wiesław Włoch (Polish Academy of Sciences Botanical Garden-Centre for Biological Diversity Conservation in Powsin). The collection of samples complies with relevant institutional, national, and international guidelines and legislation. Appropriate permission to collect plant material has been obtained. Fixed specimens used in this study were deposited in the Laboratory of Plant Anatomy collection, Polish Academy of Sciences Botanical Garden-Centre for Biological Diversity Conservation in Powsin, Poland.

Collected tissues were embedded in Epon, cut with Tesla ultramicrotome (transverse sections of a thickness ~ 3.5 μm were obtained). Then they were stained with PAS (Periodic Acid-Schiff) and toluidine blue and mounted in Euparal^30,39^. Sections were examined using bright-field microscopy (Olympus BX41 microscope). To analyse the intrusive growth of *R. pseudoacacia* initial cells, qualitative and quantitative analyses were performed. Qualitative analysis involved detailed anatomical reconstructions of tangential and radial planes, based on a series of semi-thin transverse sections.

### Measurements and data analysis

**Figure 2:**
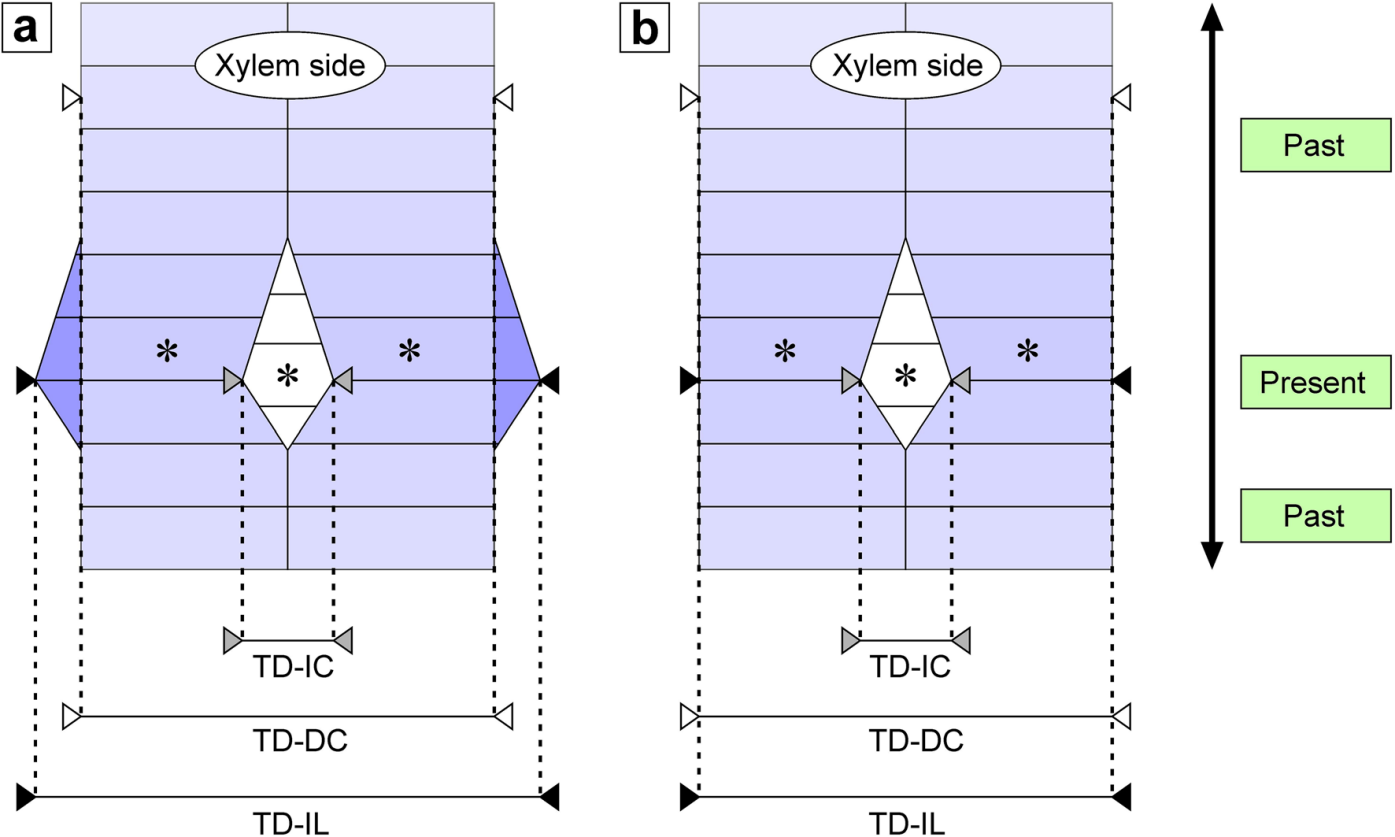
Schematic representations showing consequences of two hypotheses of intrusive growth of cambial initials. (**a**) Hypothesis no. 1: intrusive growth of a cambial initial cell has an impact on cambial circumference of a tree trunk. In this scenario (TD-DC < TD-IL) growth of an initial would be related to moving cells of neighbouring files apart in circumferential direction, thus contributing to an increase in the tangential dimension of this tissue fragment, as well as the whole circumference of a tree trunk. (**b**) Hypothesis no. 2: intrusive growth of an initial cell has no impact on the cambial circumference (TD-DC = TD-IL). Note the indication of a time aspect in reference to subsequently deposited layers of cambial derivative cells (green boxes on the right side). Radial enlargement of cells was not taken into account. Asterisks indicate cambial initials. TD-IC—tangential dimension of intrusively growing cambial initial cell measured within initial layer. TD-IL—tangential dimension of two radial files adjacent to intrusively growing initial cell together with tangential dimension of growing initial—measured in initial layer. TD-DC—tangential dimension of the same two radial files as in TD-IL, but distanced by several cells from initial layer. CorelDRAW Graphics Suite SE ver. 18.2.0.840 (https://www.coreldraw.com) was used.

Measurements were made in 60 transverse sections of the vascular cambium to test two hypotheses: (1) intrusive growth of an initial cell has an impact on the cambial circumference of a tree trunk, (2) intrusive growth of an initial cell has no impact on the cambial circumference (Fig. 2). To check whether intrusively growing initials cause an increase in tangential dimension of observed fragments of the tissue three dimensions were measured in each transverse section (Fig. 2): (1) tangential dimension of intrusively growing tip of an initial cell (marked as TD-IC; *tangential dimension-initial cell*); (2) tangential dimension of two radial files between which initial cell grows together with tangential dimension of intrusively growing tip of initial—dimension measured within the initial layer (marked as TD-IL; *tangential dimension-initial layer*); (3) tangential dimension of the same two radial files at the distance of 5–6 derivative cells from the initial layer—dimension measured on the xylem side (marked as TD-DC; *tangential dimension-derivative cells*). If it was the case that intrusive growth of a cambial initial contributes to circumferential enlargement of the vascular cambium, one should be able to observe an increase in tangential dimension of rearranging cells (tangential dimension of a growing initial cell together with tangential dimension of two neighbouring radial files would be larger than tangential dimension of these two files before occurrence of intrusive growth). Such analysis is justified because the spatial arrangement of cambial derivatives constitutes a historical record of events that took place in the initial layer in the past^6,15,24,25,28,29^. Thus, the arrangement of cells in layers located farther from the initial layer (on the xylem or phloem side), is a record of cellular events that took place in the initial layer further in the past. If we want to know what happened, let us say, ‘five periclinal divisions ago’, we should look at an adequate part of the tissue (see Fig. 2). Obviously, it should be kept in mind that a time-period between individual periclinal divisions in a given radial file of cells may differ throughout the growing season and depends upon changes in various endogenous and exogenous conditions, e.g. drought or heavy rainfall. Based on comparison of recently deposited derivatives of cambial initials (5–6 cells into the xylem) with the current situation within initial layer, and by performing precise measurements and calculations, it is possible to determine whether or not intrusively growing cell causes local increase in tangential dimension of cambial tissue. Note that examples of intrusive growth of tips of initial cells presented in Fig. 2 are hypothetical. Shapes of intrusively growing initials together with their closest derivatives may differ—from almost symmetrical to strongly asymmetrical (for more details, see Supplementary Information, Fig. S1).

Anatomical results (Table S1—Supplementary Information) were subjected to statistical analysis (Wilcoxon Matched Pairs Test; dependent model) using Statistica ver. 13.0 software (TIBCO Software Inc., Tulsa, USA). Shapiro–Wilk and Kolmogorov–Smirnov were performed to check for normality. A comparison of two groups of measurements (TD-IL and TD-DC) was performed to test for significance at the 99% confidence level to determine whether there was a significant difference between considered tangential dimensions. If the statistical analysis shows that the two groups (TD-IL and TD-DC) differ significantly, the hypothesis no. 2 is falsified. Moreover, a comparison of the minimal, maximal, and mean values calculated for all measured dimensions was made and presented as a diagram.

## Results

**Figure 3:**
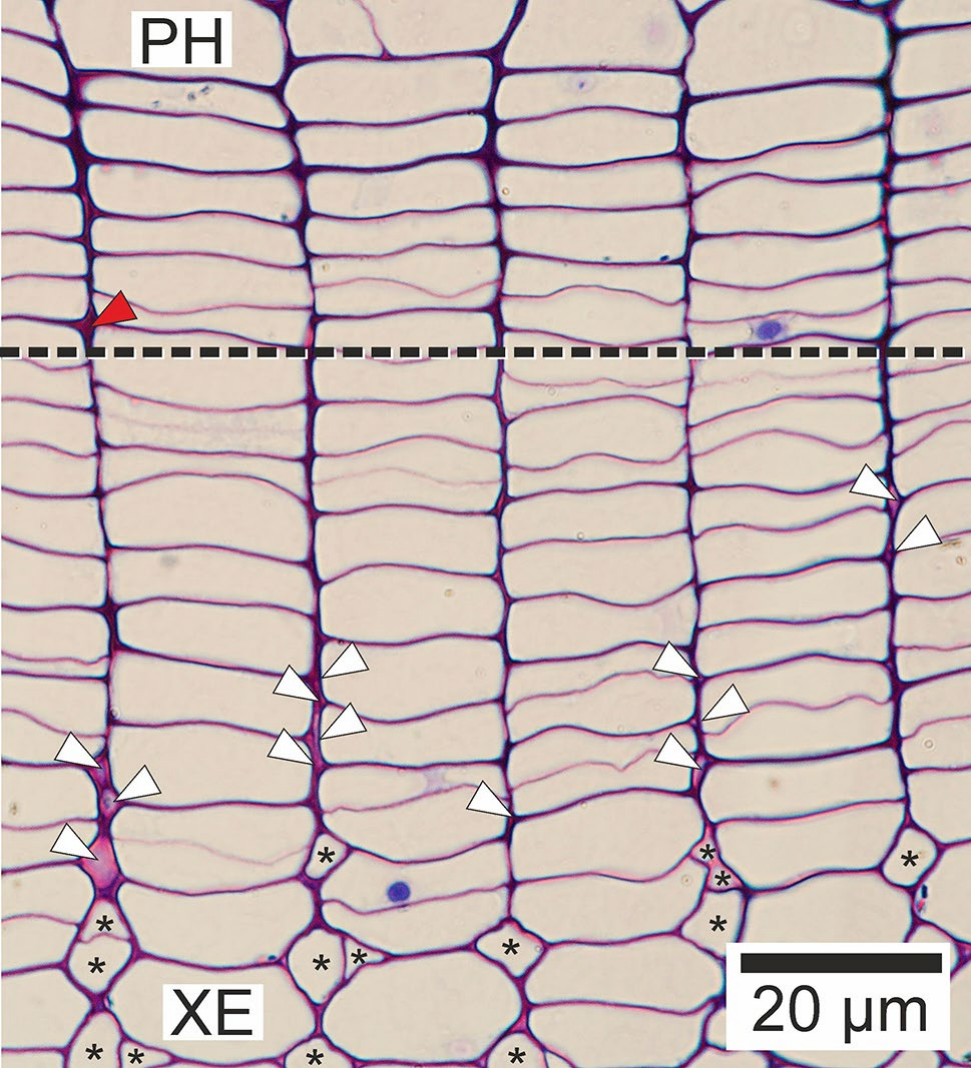
Transverse section of the vascular cambium and derivative cells collected from the tree trunk of *Robinia pseudoacacia* L. Black, dashed line indicates approximate location of cambial initial layer. PH—secondary phloem side. XE—secondary xylem side. White and red arrowheads indicate microspaces at various stages of their formation, including spots of loosening (fluidization understood as change in viscosity) of middle lamellae. Red arrowhead precedes intrusive growth of an initial cambial cell. White arrowheads precede intrusive growth of wood fibre tips. Asterisks indicate growing tips of libriform wood fibres. Adobe Photoshop CS6 Extended ver.13.0 × 64 (https://www.adobe.com), and CorelDRAW Graphics Suite SE ver. 18.2.0.840 (https://www.coreldraw.com) were used.

**Figure 4:**
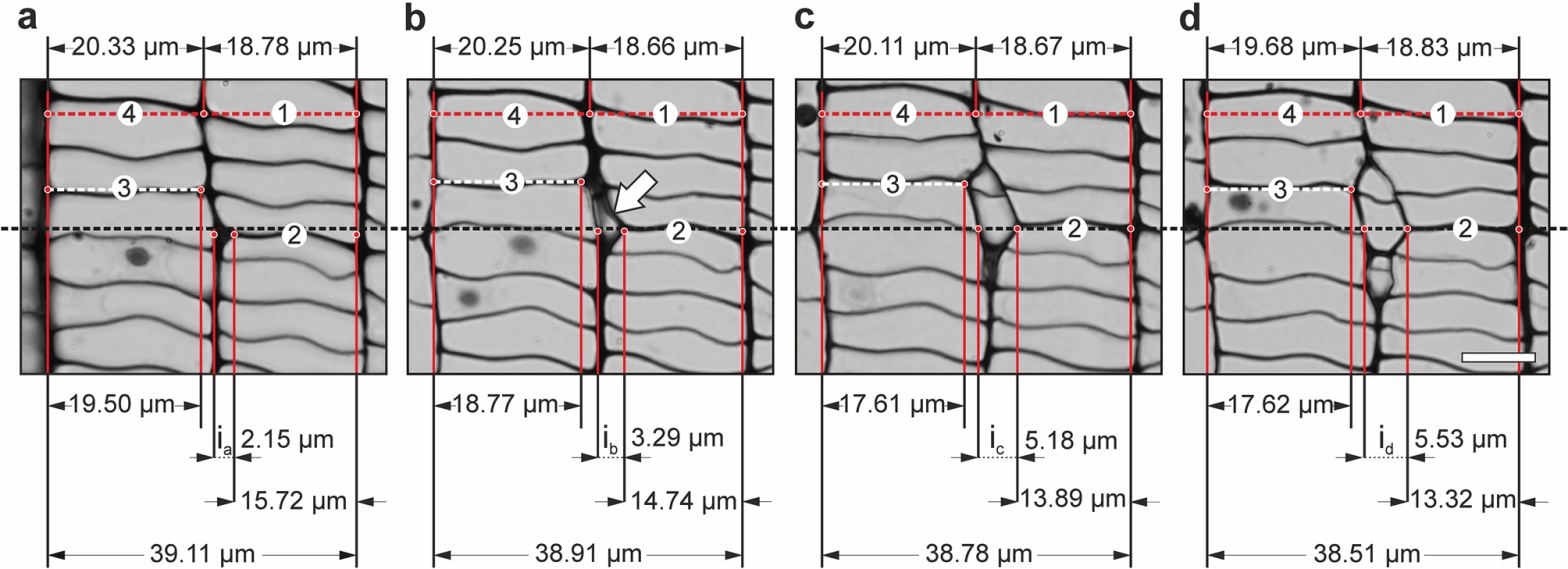
Transverse sections showing intrusively growing initial cell of the vascular cambium of *Robinia pseudoacacia* L. The four consecutive sections (**a**, **b**, **c**, **d**) are spaced from each other by ca. 3.5 μm (in axial direction). Tangential dimensions of considered elements were marked. Black, dashed line corresponds to approximate location of the initial surface. Red, dashed line goes through cambial derivative cells deposited onto the phloem side. Analysed tangential cell walls were numbered 1, 2, 3, and 4. Their length was measured without considering the curvature. i_x_ (i_a_, i_b_, i_c_, i_d_ in 4a, 4b, 4c, 4d respectively)—tangential dimension of intrusively growing tip of the cambial initial cell measured at different levels of the analysed tissue. White arrow indicates example of a slant created in the vascular cambium as a result of intrusive growth of an initial cell. Scale bar = 10 μm. Adobe Photoshop CS6 Extended ver.13.0 × 64 (https://www.adobe.com), and CorelDRAW Graphics Suite SE ver. 18.2.0.840 (https://www.coreldraw.com) were used.

Examination of tissues of *R. pseudoacacia* tree trunks allowed to state the presence of microspaces within the vascular cambium and zone of cell differentiation (Fig. 3). The formation of these spaces is a prerequisite for intrusive growth of cambial initials and wood fibre tips to occur. In the case of this study, intrusive growth of cambial initial cells took place in the storied cambium. As one can see in cross-sections, initial phases of intrusive growth of initial cells are related to the formation of obliquely oriented walls within area of rearrangement occurrence (Fig. 4).

Four transverse sections of the same storey of cambial fusiform cells are shown in Fig. 4. Selected tangential cell walls at some distance from the initial layer (walls of cambial derivative cells) were considered. The curvature of those walls was not taken into account. It allowed us to compare the length of these tangential walls (measured in transverse section) with the width of corresponding radial files near the cambial initial surface. It can be observed that reduction in the length of tangential cell wall no. 2 (Fig. 4), which is localised in the immediate vicinity of the growing tip of initial cell, progresses with an increase in tangential dimension of intruding initial cell (compare micrographs—from Fig. 4a to d). The frame of reference for this reduction in length of tangential cell wall no. 2 (related to intrusive growth of initial) is a dimension of tangential cell wall no. 1 belonging to the derivative cambial cell deposited earlier onto the phloem side. In the fragment analysed intrusive growth of the initial cell (i_x_) is not accompanied by a corresponding increase in the tangential dimension of the tissue fragment. Therefore, it can be stated that intrusive growth of an initial cell occurs between tangential cell walls of neighbouring cells and does not contribute to an increase in the circumference of the cambial cylinder.

While analysing tangential walls nos. 4 and 3, it can be noticed that the difference between their lengths is larger in Fig. 4c (20.11 μm − 17.61 μm = 2.50 μm) and 4d (19.68 μm − 17.62 μm = 2.06 μm) then in 4a (20.33 μm − 19.50 μm = 0.83 μm) and 4b (20.25 μm − 18.77 μm = 1.48 μm). The same is true for cell walls nos. 2 and 1. It means that also in the proximity of tangential cell wall no. 3 intrusive growth of cambial initial cell occurred between tangential cell walls. For this reason, it can be presumed that the initial surface of the vascular cambium, in the radial file on the left side of Fig. 4a, is a little bit more shifted (by the distance of approx. one cambial cell) towards the phloem as compared to the radial file on the right. This shows that an actual course of the initial layer does not constitute a perfectly cylindrical shape but rather a surface with slight, local deflections. This is consistent with observations made by Miodek et al.^29^.

### Quantitative analysis of intrusive growth

**Figure 5:**
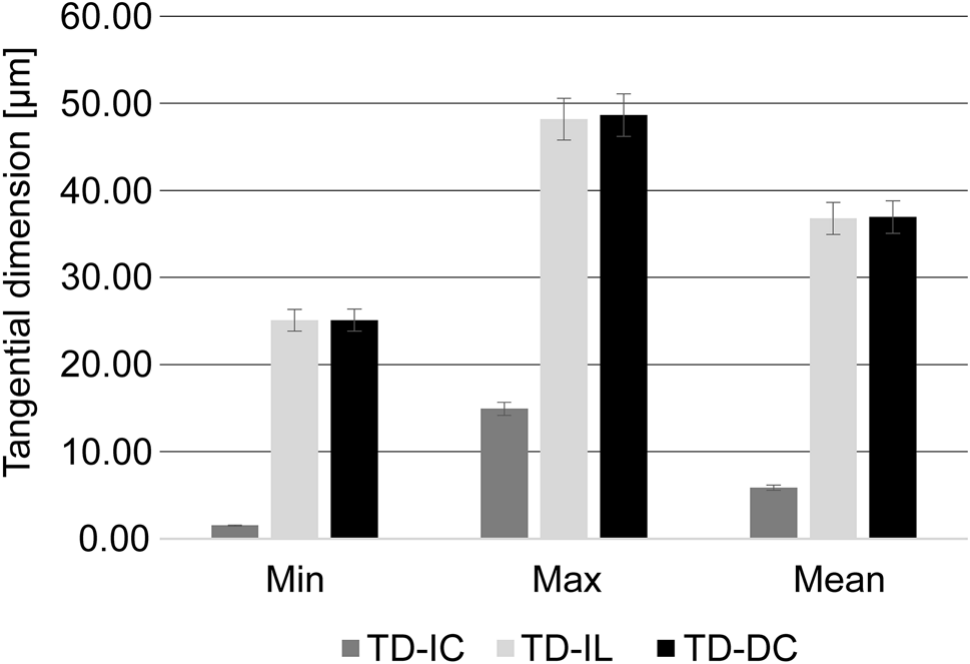
Diagram showing a comparison of the minimal (Min) and maximal (Max) values obtained, as well as mean values calculated for the dimensions measured, i.e. tangential dimension of an intrusively growing initial (TD-IC), tangential dimension of two radial files adjacent to intrusively growing initial together with tangential dimension of growing initial—measured in initial layer (TD-IL), and tangential dimension of the same two radial files distanced from initial layer (TD-DC) in *Robinia pseudoacacia*. Standard error bars (5%) are included. The diagram was based on data presented in Table 1. Adobe Photoshop CS6 Extended ver.13.0 × 64 (https://www.adobe.com), and CorelDRAW Graphics Suite SE ver. 18.2.0.840 (https://www.coreldraw.com) were used.

Measurements made for quantitative analysis showed that the mean values of TD-IL and TD-DC in 60 semi-thin sections of cambial tissue are almost identical (Table 1). Moreover, even minimal and maximal values measured in the case of TD-IL and TD-DC are very similar—see Fig. 5. Minimal values for TD-IL and TD-DC were 25.08 and 25.11 µm respectively. Maximal values for TD-IL and TD-DC were 48.17 and 48.63 µm respectively. Standard deviation for TD-IL and TD-DC was also very similar and equal to 4.98 and 4.96 µm respectively. TD-IC ranged from 1.52 to 14.93 µm, with a mean value of 5.86 µm.

**Table 1:** Minimal (Min), maximal (Max), mean values, and standard deviation (SD) calculated for tangential dimension of an intrusively growing initial cell (TD-IC), tangential dimension of two radial files adjacent to intrusively growing initial together with tangential dimension of intrusively growing tip of initial—measured in initial layer (TD-IL), and tangential dimension of the same two radial files as in TD-IL, but distanced (5–6 cells) from an initial layer (TD-DC).

Group of measurements	Min (µm)	Max (µm)	Mean (µm)	SD (µm)
TD-IC	1.52	14.93	5.86	2.52
TD-IL	25.08	48.17	36.77	4.98
TD-DC	25.11	48.63	36.93	4.96

Statistical analysis results (Wilcoxon Match Pairs Test at α = 0.01 confidence level) showed that the two considered groups, i.e. TD-IL and TD-DC, did not differ significantly from each other (Table 2). This falsifies the hypothesis no. 1 and supports the second hypothesis that intrusive growth of initial cells does not contribute to the tangential enlargement of fragments of the vascular cambium undergoing cell rearrangement, and therefore does not participate in circumferential growth of this secondary meristematic tissue.

**Table 2:** Wilcoxon Matched Pairs Test performed for tangential dimension of two radial files adjacent to intrusively growing initial together with tangential dimension of intrusively growing tip of initial—measured in initial layer (TD-IL), and tangential dimension of the same two radial files distanced from initial layer (TD-DC).

Wilcoxon matched pairs test, α = 0.01				
Pair of Variables	Valid N	T	Z	*p* value
TD-IL & TD-DC	60	789.0000	0.927562	0.353636

### Qualitative analysis of intrusive growth-reconstructions of radial and tangential planes

**Figure 6:**
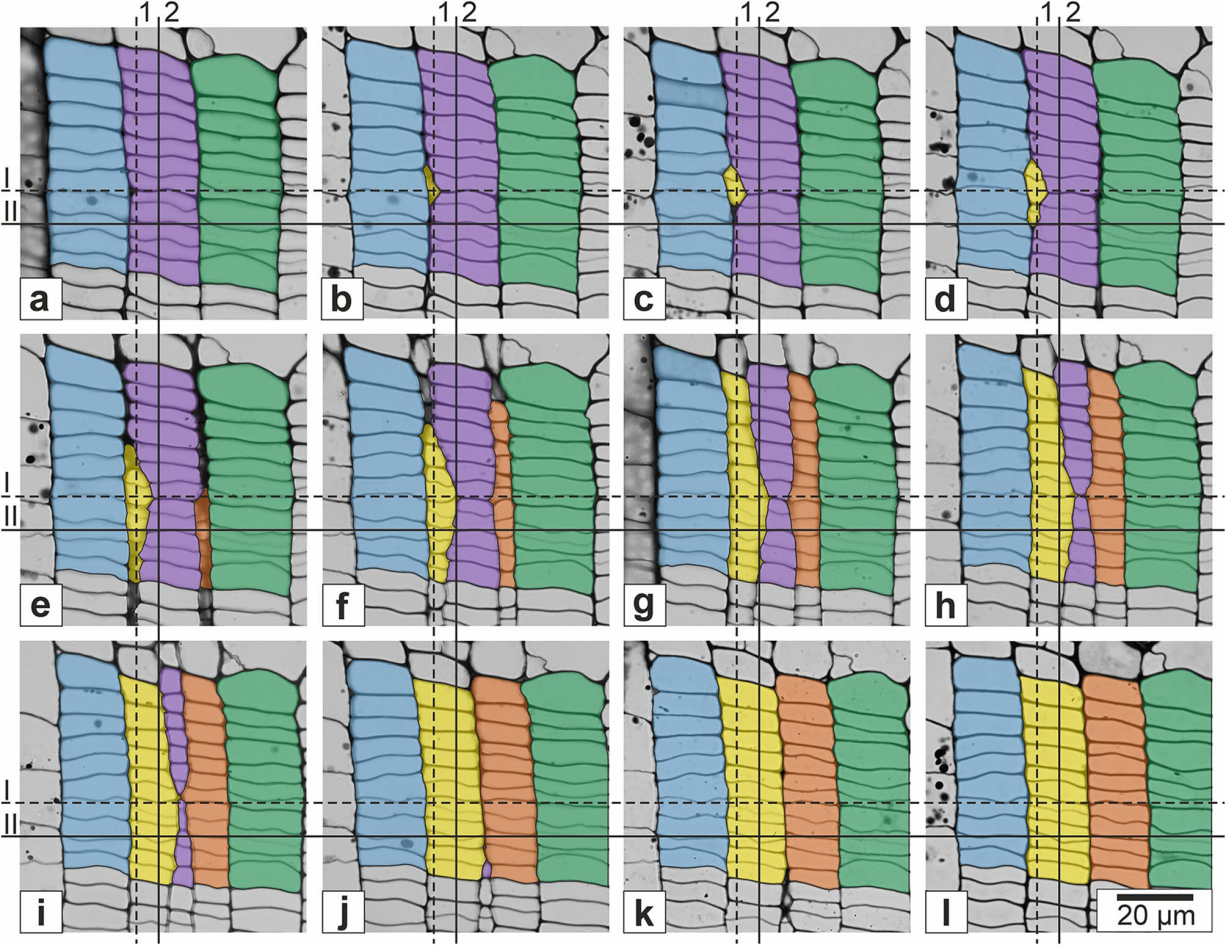
Long series of semi-thin transverse sections of the vascular cambium of *Robinia pseudoacacia* L. (**a**-**l**). Five radial files of cambial cells were distinguished. Three files in **a** are coloured as: blue, violet, and green. Files derived from intrusively growing initials are coloured in yellow and orange. Roman and Arabic numerals describe lines showing location of reconstructed tangential and radial planes respectively (see Figs. 7 and 8). Line I corresponds to approximate location of cambial initial layer. Adobe Photoshop CS6 Extended ver.13.0 × 64 (https://www.adobe.com), and CorelDRAW Graphics Suite SE ver. 18.2.0.840 (https://www.coreldraw.com) were used.

Based on a series of consecutive anatomical transverse sections of the storied vascular cambium of *R*. *pseudoacacia* a reconstruction of two radial, and two tangential planes was made. The output series is shown in Fig. 6. In 6a three neighbouring radial files were marked in blue, violet, and green. In consecutive transverse sections, one can observe increasingly advanced stages of intrusive apical growth of two initial cells originating from different stories of the vascular cambium. In 6b–d, cell rearrangement occurring due to intrusive growth of initial cell marked in yellow is visible. In 6e emergence of the second initial cell and its derivatives can also be observed (marked in orange). Note that along with the progression of intrusive growth within the yellow and orange radial files, the elimination process of the violet radial file takes place. It is worth to emphasize that intrusive growth of the initial cell of the yellow file alone does not entail tangential spacing of considered files (blue, violet, and green files), between which the growth took place. Distance between the blue and green files starts to increase only after the appearance of the orange file. It should be highlighted that the appearance of the orange file is not typical. As shown in Fig. 6 the orange file appears simultaneously in large part of the vascular cambium (closer to xylem). Usually, initial phases of intrusive growth may be recognised by characteristic slanted walls^15,28,29^. Intrusively growing initial undergoes periclinal divisions resulting in deposition of successively larger derivatives on xylem and phloem side. Simultaneous appearance of many cells of the orange file with comparable width along its entire length in the cambium seems like a rather peculiar course of events, which certainly deserves some attention in future research. In Fig. 6, changes in the shape, and dimensions of the cambial initial cell of the yellow file can be noticed—its radial dimension is initially longer than the tangential one (Fig. 6b–d). In later stages (Fig. 6e–l) tangential dimension becomes the larger one. It means that the intrusively growing tip of the initial cell has just recently started its tangential expansion, and finally would have attained a typical tangential dimension (typical width of cambial cells in this species).

**Figure 7:**
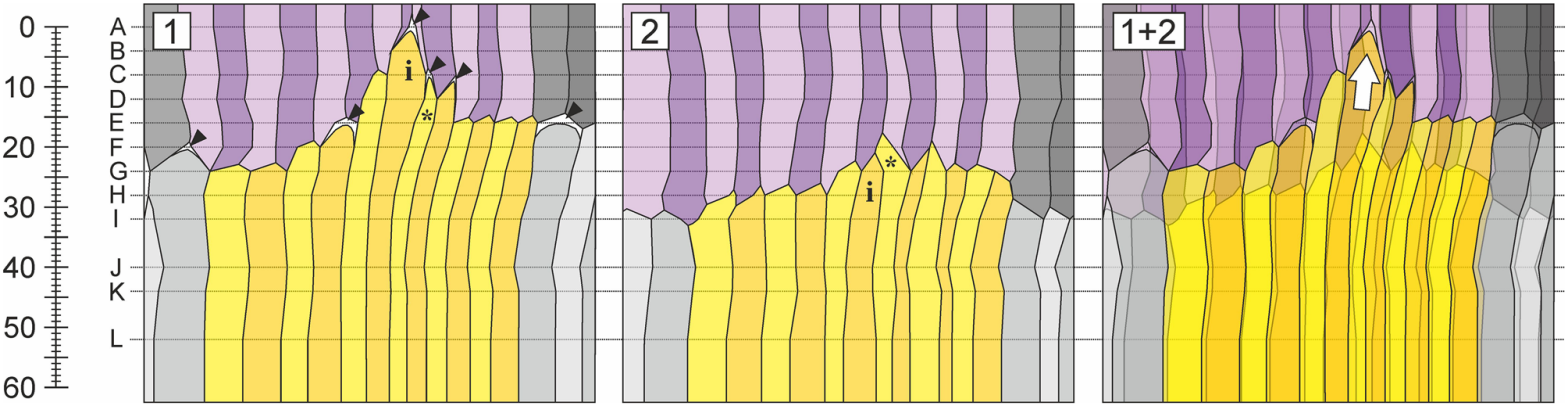
Anatomical reconstructions of radial planes of the vascular cambium of *Robinia pseudoacacia* L. Scale on the left is expressed in μm. (**1**) Reconstruction of radial plane 1 shown in Fig. 6. (**2**) Reconstruction of radial plane 2 shown in Fig. 6. (**1 + 2**) Superposed radial reconstructions 1 and 2. i—cell considered as cambial initial in plane 1. *—cell considered as cambial initial in plane 2. Black arrowheads indicate microspaces, which are usually formed prior to intrusive growth of a cell. White arrow indicates the direction of intrusive elongation of cell i. CorelDRAW Graphics Suite SE ver. 18.2.0.840 (https://www.coreldraw.com) was used.

Reconstructions of the radial planes are shown in Fig. 7. Separation of tangential cell walls in the vicinity of intrusively growing cell marked with ‘i’ can be observed. The occurrence of microspaces, which precede intrusive growth of cambial cells or their derivatives, is visible in plane no. 1 (Fig. 7; black arrowheads). Note that the cell marked with ‘*’ reaches the highest axial position in plane no. 2. This means that in plane no. 2 the functional role of an initial can be assigned to this specific cell. However, in plane no. 1, cell marked as ‘i’ plays the role of an initial. This peculiar detail seems to be of importance in determining the status of cambial initial cell.

**Figure 8:**
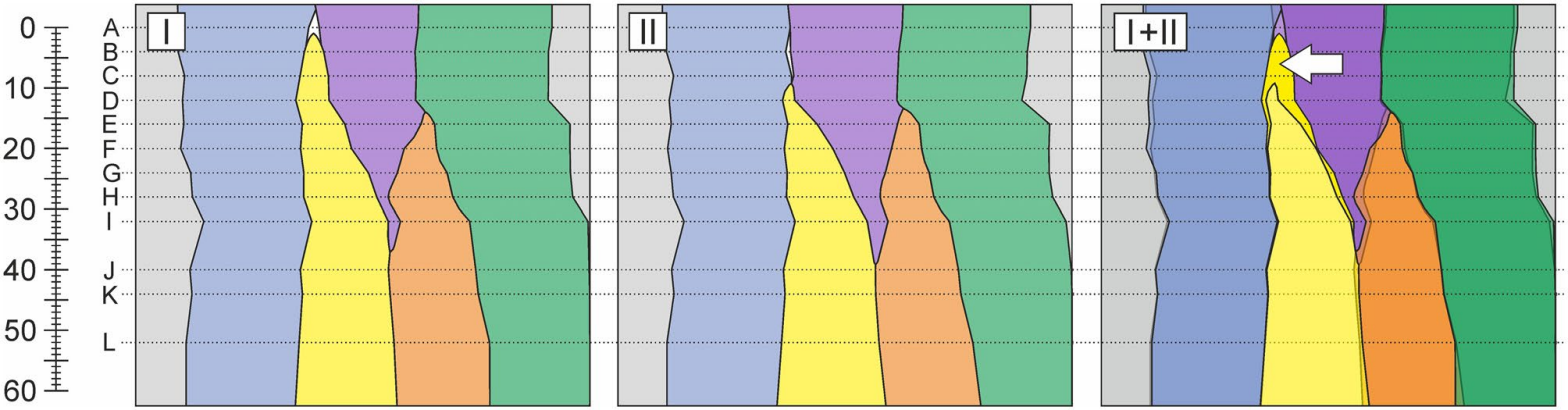
Anatomical reconstructions of tangential planes of the vascular cambium of *Robinia pseudoacacia* L. Scale on the left is expressed in μm. (**I**) Reconstruction of tangential plane I indicated in Fig. 6. (**II**) Reconstruction of tangential plane II indicated in Fig. 6. Plane I is localised in close vicinity of the cambial initial surface. Plane II is localised closer towards the xylem side. (**I + II**) Superposed reconstructions of tangential planes I and II, showing that intrusive growth of the initial cell does not contribute to the increase in tangential dimension of analysed fragment of the tissue, and therefore do not increase the circumference of cambial cylinder in examined tree trunk. It also indicates that growth of the initial cell occurred between tangential cell walls of neighbouring cells. White arrow indicates part of the cell which grew intrusively. CorelDRAW Graphics Suite SE ver. 18.2.0.840 (https://www.coreldraw.com) was used.

Reconstructions of tangential planes I, and II (indicated in Fig. 6) are shown in Fig. 8. Superposed planes I and II (marked as I + II) reveal the range of intrusive elongation of the cambial initial cell shown in yellow. The observed extent of intrusive growth measured approx. 10 μm. It is worth acknowledging that in tangential plane II (Fig. 8), on the level of transverse section B, there is a small spacing between blue and violet radial files, which may be interpreted as a microspace. Examples of such microspaces have already been shown in Fig. 3 (transverse view).

## Discussion

### Increase in the circumference of vascular cambium versus intrusive growth

In case of the storied vascular cambium, an assumption that the mechanism of increase in cambial circumference involves symplastic growth of cambial cells which undergo longitudinal anticlinal divisions predominates in the literature^9,10,12,13^. This assumption was related to observations of the number of anticlinal divisions in relation to the increase in cambial circumference. In the storied cambium, longitudinal divisions are the most abundant type of anticlinal divisions^1,40–42^. The number of such divisions, together with symplastic growth of resultant sister cells, were considered to compensate for the increase in cambial circumference^24^. Therefore, in the storied cambium, intrusive growth was neither theoretically nor practically required to explain the mechanism of radial growth of cambial cylinder—as opposed to the non-storied cambium^1,7^, in which intrusive growth is characterized by a greater range ^1^. Nonetheless, intrusive growth also occurs in storied cambia^15^. If intrusive growth of cells is thought to affect cambial circumference leading to its increase in the nonstoried cambium, then why it would not cause the same effect in the storied cambium? The analysis of numerous transverse sections of the storied cambium of *R. pseudoacacia* has not confirmed the hypothesis that neighbouring initials move apart in a circumferential direction after the appearance of an intrusively growing tip of the initial cell between them. Anatomical reconstructions of cambial tangential surfaces (Fig. 8) also do not confirm this statement, as superposing two tangential planes (I and II—spaced from each other by several µm in radial direction) revealed that intrusive growth of the cell tip (indicated by white arrow in Fig. 8) is not related to moving neighbouring cells (between which the intrusion occurred) apart. Statistical analysis showed no significant differences between measured TD-IL and TD-DC. This supports the conclusions drawn from anatomical observations. Our results are consistent with previous reports on the mode of intrusive growth of cambial initials in dicotyledonous species with storied structure of the vascular cambium like *L. sericeus*^25^, *T*. *cordata*^15^ and *W*. *floribunda*^15,24^, and coniferous species with nonstoried cambium like *P. abies*^30^, and *P. sylvestris*^15,26–28^.

Interestingly, an assumption that there exist two different mechanisms of increasing the cambial circumference—one for nonstoried and other for storied cambium structure—leads to the conclusion that during formation of the storied structure of cambium from the nonstoried one, a mechanism of growth of cambial circumference would have to change^19^. Location of intrusive growth of initials between tangential walls of neighbouring cells^15,29^ seems to be a solution to the problem of existence of two different mechanisms of cambial growth, as well as the issue of changing mechanism of cambial increase in circumference during the formation of the storied structure within individual tree^19^. According to the results of the latest observations, it seems that a mechanism of increase in cambial circumference is the same for both nonstoried and storied cambia and stems from the symplastic growth (summarized in: Miodek et al.^29^). The role of intrusive growth of initial cells, which occurs independently of periclinal divisions of the mother cells, would be universal for both types of cambia and result in a gradual rearrangement of the initial surface^22,24–26^. A current arrangement of the cambial initials is constantly recorded in the newly formed layers of cambial derivatives deposited on the xylem or phloem side^6,29^.

Together with the progression of the intrusive growth of an initial cell, separated tangential cell walls between which intrusive growth occurs first become obliquely and then radially oriented. At the stage of transformation of tangential walls into oblique ones characteristic slants are visible in the cross-sections of the vascular cambium^6,15,29^ (Fig. 4). Our results indicate that it is necessary to re-examine the role and type of mechanical stresses operating in the vascular cambium^13,43,44^, since it is difficult to explain intrusive growth of cambial initials between tangential walls of neighbouring cells, while simultaneously assuming that they are compressed in radial direction. The mode of growth of initials observed in this study becomes easy to explain through the lenses of the new hypothesis of radial growth of broadleaved trees^5,45–47^. Occurrence of radial tensile stress postulated by this hypothesis (occurring periodically—in diurnal cycles—throughout the growing season), which is generated in the vascular cambium by the differential swelling of phloem and xylem^5,45^ results in: (I) formation of microspaces between tangential walls of cells; (II) intrusive growth of initials between separated tangential walls of neighbouring cambial cells^46,47^.

In summary, our results support a view that intrusive growth of initial cells occurs between tangential cell walls of neighbouring initials and their immediate derivatives^16,28^. Thus, it can be stated that this type of growth is associated with the rearrangement of cambial cells^6,28^, and not with the increase in cambial circumference^29^.

### Status of initial cell

Radial plane reconstructions revealed parts of two cells belonging to the same radial file acting as initials at the same moment (given that an initial cell is recognised as the one elongated the most in axial direction—as a consequence of intrusive growth). Currently, there is an agreement that in each radial file of cells, only one cell is functioning as an initial, thus being responsible for the deposition of derivatives differentiating into both xylem and phloem elements^1,7,9,13,40,48–50^. It seems that reconstructions of radial planes of *R. pseudoacacia* cambium allowed us to capture the moment of transition of the initial status from one cell to another, contiguous cell of the same radial file. Cell ‘i’ gained the status of the initial cell in plane 1. Note that the microspace was formed above cell ‘i’, not above cell ‘*’. Thus, the microspace indicates a shift in growth dynamics towards plane 1 at that time. In 1980 Catesson stated that it cannot be ruled out that under certain conditions the initial status of a cell can be transferred from one to another cell (the closest cell in considered radial file)^40^. The author also emphasized that change in the cell status (derivative cell vs. initial cell) may be associated with ensuring greater responsiveness to changes in stress conditions^40^. As shown by the most recent studies, the determination of an initial cell in the functioning secondary meristem is reversible—the initial cell may lose its status by being eliminated from an initial surface. The eliminated initial cell will eventually differentiate into xylem or phloem cell after undergoing several unequal periclinal divisions^15,16,27^. A scenario in which the status of an initial cell is transferred from one cell to another within one radial file is easy to explain by the new hypothesis of radial growth of broadleaved trees^5,46,47^. When and how certain cells divide may primarily depend on the mechanical stress distribution. This type of relationship was indicated by the studies of Lintilhac and Vesecky^51^ and Lynch and Lintilhac^52^. Stress (the resultant force acting on selected cells or their fragments) can also determine which cambial cells will act as initials, and thus be subjected to constant periclinal divisions. Moreover, various configurations of compressive and tensile stresses (also their varied intensity in time) may determine the type of cell divisions (anticlinal longitudinal, oblique, transverse; periclinal, uneven periclinal). Such a system, based on the field of mechanical stress, would be characterized by high precision, almost instantaneous signal perception, and thus (directly and indirectly) a fast reaction to changes in temperature, osmotic potential, hydrostatic pressure, cytoskeleton arrangement, hormone gradients, etc. From an ontogenetic and evolutionary perspective, it would entail a substantial adaptive potential, due to the fast signal transduction, which would integrate versatile input signals affecting cell walls/plasma membranes. This seems to be of crucial importance in determining the status of an initial cell. Consequently, it can be expected that a dynamically adapting field of mechanical stress operates in the vascular cambium and surrounding tissues.

## Conclusions

It was shown with both quantitative, and qualitative analysis that intrusive growth of initial cells has no effect on the circumference of the cambial cylinder. Our results support the assumption that intrusive growth of an initial cell occurs between tangential walls of another initial cell and its neighbouring derivative. Anatomical reconstructions of radial planes of the vascular cambium allowed us to capture the moment of transition of initial status from one cell to another, contiguous cell of the same radial file. This is a temporal state, preceding unequal periclinal divisions of these two resultant initials (one growing intrusively and second undergoing elimination). Our results can be explained considering the mechanical conditions in the vascular cambium, mainly periodical occurrence of radial tensile stress.

## Supplementary Information

Supplementary Information.

## Data Availability

All data generated or analysed during the study are included in this published article and its Supplementary Information.
