# Supplementary material for: Intrusive growth of initials does not affect cambial circumference in Robinia pseudoacacia
Source: Sci Rep. 2022 May 6;12:7428. doi: 10.1038/s41598-022-11272-y (PMC9076624; doi:10.1038/s41598-022-11272-y)
Supplement: Supplementary file 1 — Supplementary Information. [file 41598_2022_11272_MOESM1_ESM.pdf]

Intrusive growth of initials does not affect cambial circumference in *Robinia pseudoacacia*

Adam Miodek[1,2,*], Aldona Gizińska[1,2], Wiesław Włoch[1] & Paweł Kojs[1]

[1]Polish Academy of Sciences Botanical Garden – Centre for Biological Diversity Conservation in Powsin, Prawdziwka 2, 02-973 Warsaw, Poland. [2]Institute of Biology, University of Opole, Oleska 22, 45-052 Opole, Poland.

Supplementary Information

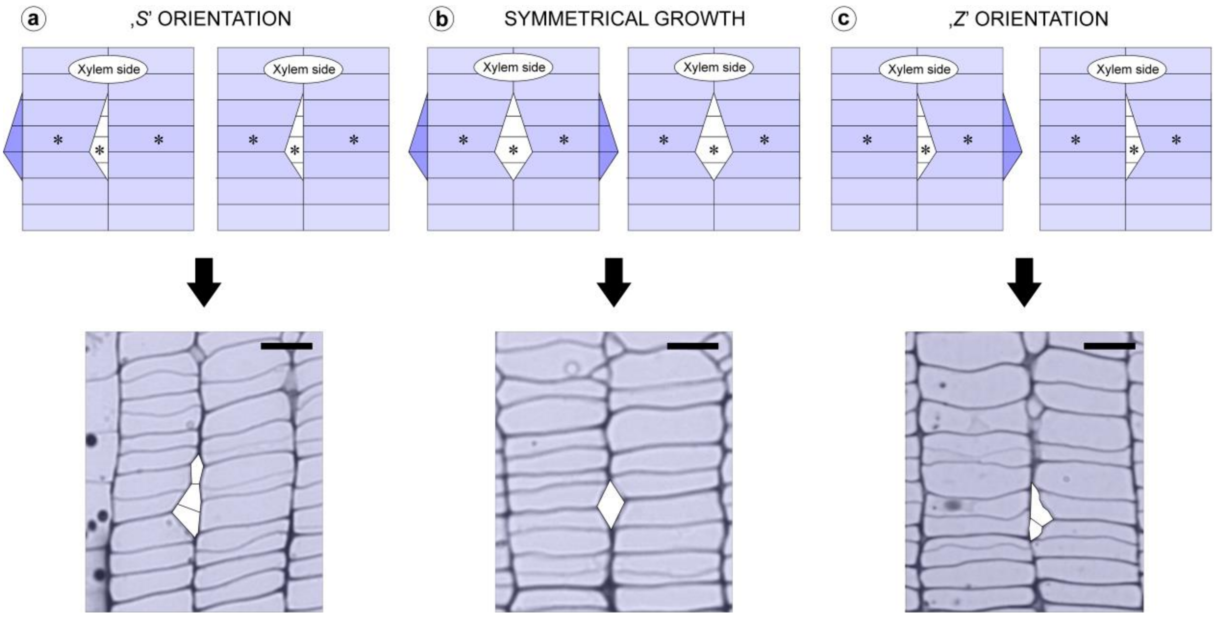

Figure S1. Schematic representations of two tested hypotheses with different shapes of intrusively growing initials and their closest derivatives as well as micrographs of real tissues corresponding with these shapes. Hypothesis no. 1 (intrusive growth of an initial cell has an impact on cambial circumference of a tree-trunk) is shown on the left side of each figure (**a**, **b** and **c**). Hypothesis no. 2 (intrusive growth of an initial cell has no impact on the cambial circumference) is shown on the right side of each figure (**a**, **b** and **c**). **a** Asymmetrical intrusive growth of an initial cell showing *S* orientation – inclined towards left. **b** Symmetrical growth of an initial cell. **c** Asymmetrical intrusive growth of an initial cell showing *Z* orientation – inclined towards right. Note that shapes of intrusively growing initials and their closest derivatives do not affect the measurement method. Scale bar = 10 μm. Adobe Photoshop CS6 Extended ver.13.0 x64 (https://www.adobe.com), and CorelDRAW Graphics Suite SE ver. 18.2.0.840 (https://www.coreldraw.com) were used.

No.	TD-IC (µm)	TD-IL (µm)	TD-DC (µm)	No.	TD-IC (µm)	TD-IL (µm)	TD-DC (µm)	No.	TD-IC (µm)	TD-IL (µm)	TD-DC (µm)
1	5.01	43.49	42.22	21	6.96	35.90	35.33	41	7.71	41.96	40.91
2	3.55	25.08	25.11	22	5.25	33.27	33.39	42	14.45	44.48	43.84
3	3.06	26.07	26.57	23	7.76	41.49	41.70	43	3.68	38.51	38.59
4	5.71	31.61	30.44	24	6.66	40.54	40.13	44	4.69	30.50	30.10
5	5.60	32.61	32.68	25	6.59	40.69	40.65	45	6.10	30.56	29.79
6	4.24	36.84	35.14	26	5.38	40.07	40.01	46	4.39	32.37	32.73
7	4.02	39.06	38.53	27	4.87	35.98	35.65	47	5.53	43.82	43.84
8	4.26	35.02	34.31	28	6.34	37.21	38.86	48	6.82	34.57	34.48
9	6.84	38.22	37.63	29	3.80	43.01	43.36	49	5.59	34.35	36.03
10	7.30	38.14	38.32	30	4.09	33.37	33.83	50	10.91	34.59	36.17
11	8.72	38.39	37.85	31	5.35	34.81	35.54	51	4.90	34.62	34.59
12	5.23	40.23	39.75	32	1.52	26.07	26.09	52	4.53	36.99	35.58
13	2.98	44.27	43.82	33	10.82	28.96	29.14	53	4.48	36.28	37.12
14	3.53	30.12	32.65	34	4.10	31.37	31.20	54	5.93	39.57	39.97
15	4.01	35.18	35.15	35	5.95	30.49	31.25	55	3.38	38.76	38.94
16	6.85	32.94	34.70	36	6.55	34.91	36.74	56	9.65	46.21	46.49
17	4.79	37.85	37.81	37	5.03	36.16	34.87	57	3.77	48.17	48.63
18	4.45	42.39	43.87	38	3.84	37.10	39.58	58	7.78	40.59	40.33
19	4.75	41.20	41.97	39	8.99	34.46	33.66	59	8.77	38.99	39.08
20	4.96	42.49	43.28	40	3.66	35.09	36.59	60	14.93	38.16	39.12

Table S1. Tangential dimension of intrusively growing tip of an initial cell measured within an initial layer (TD-IC; *tangential dimension-initial cell*), tangential dimension of two radial files between which initial cell grows together with tangential dimension of intrusively growing tip of initial – dimension measured within the initial layer (TD-IL; *tangential dimension-initial layer*), and tangential dimension of the same two radial files at the distance of 5–6 derivative cells from the initial layer – dimension measured on the xylem side (TD-DC; *tangential dimension-derivative cells*).
